# Computational identification of surrogate genes for prostate cancer phases using machine learning and molecular network analysis

**DOI:** 10.1186/1742-4682-11-37

**Published:** 2014-08-23

**Authors:** Rudong Li, Xiao Dong, Chengcheng Ma, Lei Liu

**Affiliations:** 1 Key Laboratory of Systems Biology, Shanghai Institutes for Biological Sciences (SIBS), Chinese Academy of Sciences (CAS), Shanghai 200031, China; 2 Shanghai Center for Bioinformatics Technology (SCBIT), Shanghai 201203, China; 3 Institutes for Biomedical Sciences, Fudan University, Shanghai 200031, China

## Abstract

**Background:**

Prostate cancer is one of the most common malignant diseases and is characterized by heterogeneity in the clinical course. To date, there are no efficient morphologic features or genomic biomarkers that can characterize the phenotypes of the cancer, especially with regard to metastasis – the most adverse outcome. Searching for effective surrogate genes out of large quantities of gene expression data is a key to cancer phenotyping and/or understanding molecular mechanisms underlying prostate cancer development.

**Results:**

Using the maximum relevance minimum redundancy (mRMR) method on microarray data from normal tissues, primary tumors and metastatic tumors, we identifed four genes that can optimally classify samples of different prostate cancer phases. Moreover, we constructed a molecular interaction network with existing bioinformatic resources and co-identifed eight genes on the shortest-paths among the mRMR-identified genes, which are potential co-acting factors of prostate cancer. Functional analyses show that molecular functions involved in cell communication, hormone-receptor mediated signaling, and transcription regulation play important roles in the development of prostate cancer.

**Conclusion:**

We conclude that the surrogate genes we have selected compose an effective classifier of prostate cancer phases, which corresponds to a minimum characterization of cancer phenotypes on the molecular level. Along with their molecular interaction partners, it is fairly to assume that these genes may have important roles in prostate cancer development; particularly, the un-reported genes may bring new insights for the understanding of the molecular mechanisms. Thus our results may serve as a candidate gene set for further functional studies.

## Background

Prostate cancer is one of the most frequently-occurred malignant diseases affecting human health and life qualities [1]. In this cancer, metastasis (i.e. tumor cells escaping from the primary tissue and eventually colonizing a distant site) reflects the most adverse phase, which commonly results in disruption of a complex set of biological processes, causing severe bone pain and spinal cord complications [2,3]. Due to the heterogeneity of the disease, there are currently no reliable morphologic features or genetic/genomic biomarkers that can effectively discriminate tissue-confined primary and/or metastatic tumors, thus less is known for the mechanisms underlying the development of metastatic disease.

Many efforts have been devoted to revealing the molecular mechanisms underlying the disease progression and/or identifying genetic/genomic surrogates for the tumor phenotypes. In most of the studies, the phenotype of a tumor is defined by its phase [4,5]; and identification of molecular surrogates underlying the different tumor phases is facilitated by classification of samples from the respective phases (i.e. normal prostate, primary tumor, and metastatic tumor). Since the different phases constitute the process of disease progression, the surrogates (i.e. set of genes) that distinguish the phases (or classify samples from different phases) would certainly provide insights for understanding the molecular mechanisms of disease progression. For prostate cancer, gene expression microarray studies have characterized expression profiles of primary cancers, metastatic cancers and normal tissues [6-8]; in some cases, correlations between gene expressions and cancer phases have been revealed [9]. The studies have further led to the finding that differential gene expression profiles hold for metastatic androgen ablation resistant prostate cancer (AARPC) and androgen-dependent metastatic cancers [10]. In general, these results have gained important insights about metastatic prostate cancer, regarding to the changes in expressions of genes involved in various biological processes, e.g. signal transduction, cell cycle, cell adhesion, migration and mitosis, etc. [11,12]. Nonetheless, one important problem remains: previous studies describe the correlations of expression profiles and disease phases in terms of hundreds of genes, whereas they seldom provide a convenient molecular measure (i.e. minimum predictor gene set) for accurate classification of prostate cancer phases, especially with respect to metastasis. Such a predictor gene set would be a better highlight for the mechanisms of prostate cancer.

To address this issue, we herein adopt a two-step pipeline widely-used in previous studies, which includes machine learning to identify disease-related genes and pathway analysis to reveal molecular interactions among the genes [13-16]. First, we utilize the machine learning strategy for accurate classification of prostate cancer phenotypes based on gene expression microarray data. Specifically, we use the minimum redundancy – maximum relevance method (mRMR), a robust method with a broad spectrum of applications [13,17], to serve our goal of identifying a largest-parsimony (i.e. minimum) surrogate (i.e. gene set) for prostate cancer phases. Moreover, in order to focus more on the issue of metastasis, we not only consider gene expression data of normal and (tissue-confined) primary prostate tumor tissues [7], but also include a previously published dataset of metastatic tumor samples (i.e. tissue samples excluding potentially uninformative stromal genes) in our study [11].

Furthermore, genes/proteins usually co-function with their interaction partners; thus molecular interaction partners of disease-related genes are also candidates for further studies. For this purpose, we pinpoint the identified surrogate genes in a molecular interaction network constructed based on STRING (Search Tool for the Retrieval of Interacting Genes), which is a database providing resources of molecular interaction information [18]; and we then identify by the shortest-path analysis a set of potential co-acting factors, which may serve as candidate causal genes for further experimental studies.

## Materials and methods

### Data source

The gene expression dataset was adopted from a research on prostate cancer by Chandran *et al*. [11]. The data were with the Affymatrix GPL92 platform and generated from 167 samples, which contained 77 normal tissues (NTs, including both normal prostate tissues free of pathological alterations from organ donor and normal tissues adjacent to tumor), 66 primary prostate tumors (PTs) and 24 metastatic tumors (MTs). All tissue samples were acquired from the Health Sciences Tissue Bank of the University of Pittsburgh Medical Center under stringent Institutional Review Board guidelines with appropriate informed consent [11]. The data were downloaded from NCBI Gene Expression Omnibus (GEO) with accession number GSE6919. The normalized expression data were obtained directly from the GEO website, in which the data were normalized by global scaling and analyzed with Microarray Suite version 5.0 (MAS 5.0) using Affymetrix default settings. In our machine learning procedure, we did not combine the expressions from probes to genes; instead we obtained results at the probe level directly. We focused our analysis on probes corresponding to protein coding genes.

### Algorithm of mRMR & prediction engine

The minimum redundancy – maximum relevance (mRMR) algorithm was utilized herein to select surrogate genes for prostate cancer progression. The major steps of mRMR implementation were the same as we previously described [13]. The algorithm aimed to balance features’ relevance to the prediction target and the redundancy between features. Both relevance and redundancy were quantified with mutual information (MI), estimated as,

(1) $$I\left(x,y\right)=-\frac{1}{2}\ln\left(1-\rho {\left(x,y\right)}^{2}\right)$$

where *I* represented the MI and *ρ* was the correlation coefficient between the variables *x* and *y*.

First, assume that *y* was the input variable, and *X* = { *x*_*1*_, …, *x*_*n*_ } was the set of input features. Given *x*_*i*_ as the feature with the highest MI with the input variable, the feature set (*S*) at the current step was then initialized by *x*_*i*_. Second, we selected the feature *x*_*j*_ with the best balance between highest relevance and lowest redundancy and added it to *S*. It was achieved by maximizing the score *q* as follows,

(2) $$q=I\left(x_{j},y\right)-\frac{1}{\left|S\right|}\sum_{x_{k}\in S}I\left(x_{j},x_{k}\right)$$

We repeated the above steps until a desired solution length was reached. The mRMR algorithm was implemented using the R package “mRMRe” [19].

We predicted the phenotype of an individual in three ways: 1) the phenotype of its nearest neighbor; 2) the most-occurring phenotype of its five nearest neighbors; 3) the phenotype of its nearest clustering center of each phenotype group (for detailed results, see Additional file 1: Table S1). According to Chou *et al*.’s studies [17,20], the distance between two individuals was calculated as follows,

(3) $$d\left(i_{1},i_{2}\right)=1-\frac{e_{1}\cdot e_{2}}{\left|e_{1}\right|\cdot \left|e_{2}\right|}$$

where *d* was the distance, *i*_*1*_ and *i*_*2*_ were two samples, and *e*_*1*_ and *e*_*2*_ were the vectors of selected features of *i*_*1*_ and *i*_*2*_, respectively.

### Validation & incremental feature selection

We used jackknife validation to estimate the prediction accuracy of the selected features. The advantages of jackknife comparing with other validation methods, such as independent-dataset validation and sub-dataset validation, were discussed previously [17,20]. In jackknife validation, given *X* samples of a known outcome variable and *N* selected features, for each sample we compared the known outcome with an estimated outcome, which was computed based on the rest *X* - 1 samples. We defined the accuracy of a prediction using the following formula,

(4) $$\mathrm{Accuracy}=\frac{\mathrm{TP}+\mathrm{TN}}{\mathrm{TP}+\mathrm{TN}+\mathrm{FP}+\mathrm{FN}}$$

where *TP*, *TN*, *FP* and *FN* represented the numbers of true positives, true negatives, false positives and false negatives, respectively.

**Figure 1:**
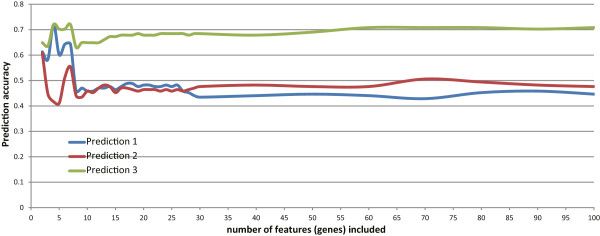
**IFS curves determining the number of features.** We used the IFS curves to determine the number of features used in mRMR computation. “Prediction 1”, “Prediction 2” and “Prediction 3” refer to the three prediction approaches, a vote of the top five nearest neighbors, the first nearest neighbor and nearest clustering center of each phenotype group, seperately. It is noted that when using four genes, the prediction accuracy reaches the maximum.

Furthermore, Incremental Feature Selection (IFS) was used to determine the number of features for optimal prediction (Figure 1). As previously described [13], for *N* = 1 to 400 required number of features, each feature set was computed by mRMR and the prediction accuracy was estimated using Jackknife validation. The set with the best prediction accuracy and smallest feature number was regard as the final feature set. In this study, a set with four genes was chosen and its prediction accuracy is 0.7202.

### Molecular interaction network & shortest-path analysis

**Figure 2:**
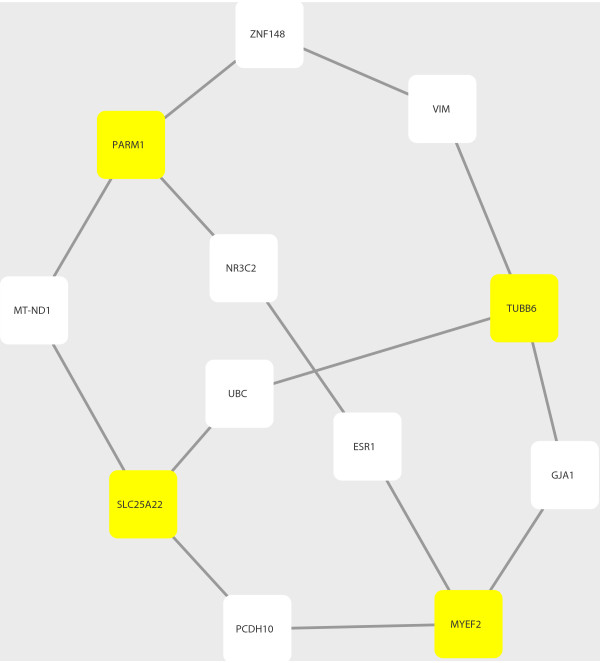
**PPI network of shortest**-**paths among the four mRMR**-**identified genes.** We identified the shortest-paths between each pair of the four mRMR-selected genes in the backgroud molecular interaction (PPI) network constructed based on STRING. Symbols of proteins and their corresponding genes are used interchangably herein. Rectangles colored in yellow are the four mRMR-selected genes/proteins; the others represent interaction parnters locating on some shortest-paths among them.

To reveal possible functional implications of the mRMR-selected genes, we explored the shortest-paths among the genes in a background molecular network constructed using the protein-protein interaction (PPI) data from STRING database (version 9.1) (http://string-db.org) [18]. To identify the shortest-path between two genes/proteins, we used Dijkstra’s algorithm and implemented it in the R package “igraph” [21]. The resulting sub-network of PPIs representing the shortest-paths among the four mRMR-selected genes (Figure 2) was visualized using Cytoscape (version 3.0.1) [22].

### GO and KEGG pathway annotation

We carried out functional annotation for all the genes identified by mRMR and shortest-path analysis based on GO and KEGG pathways. The functional annotations were implemented using the web service of DAVID tools (version 6.7) [23], by which existence of gene enrichments to certain functional modules/pathways could also be observed.

## Results

### A set of four genes presents the best accuracy for predictions of NTs vs. MTs and PTs vs. MTs

In implementation of mRMR, we consecutively tested the predictor with one feature (probes of gene expression array), two features, three features, etc., and the IFS result was provided in Figure 1. In the IFS curves, X-axis is the number of probes used for classification and Y-axis is the prediction accuracy (of the nearest-neighbor algorithm evaluated by the Jackknife validation). As shown, the accuracy for classification of NTs vs. MTs and PTs vs. MTs reaches the maximum when only four features are included, corresponding to four genes annotated in the Ensemble Biomart database (*TUBB6*, *MYEF2*, *PARM1*, *SLC25A22*). We list the genes in Table 1 and their respective expression levels in NT, PT and MT in Additional file 2: Table S2; we specifically discuss the functions of the genes in later sections.

**Table 1:** The four genes identified by mRMR

**Probe ID**	**Ensembl gene ID**	**Ensembl protein ID**	**Gene symbol**	**Gene function**
43355_s_at	ENSG00000176014	ENSP00000318697	TUBB6	Microtube formation; gap junction (intercellular communication)
55458_at	ENSG00000104177	ENSP00000316950	MYEF2	Myelination repression
54033_at	ENSG00000169116	ENSP00000370224	PARM1	Telomerase activity upregulation; prostatic cancer cell immortalization
52890_at	ENSG00000177542	ENSG00000177542	SLC25A22	Mitochondrial carrier; energy metabolism

### A PPI sub-network provides additional insights for the prostate cancer-related genes

Furthermore, we constructed an molecular interaction network with the PPI data from STRING. We traversed all pairs of any two genes from the four surrogate (protein coding) genes identified by mRMR as described above; and we then calculated the shortest paths between any pair of two proteins/genes using the Dijkstra’s algorithm. We eventually obtained a sub-network of STRING PPIs that contained all these shortest paths (Figure 2). There are a total of 14 protein-protein interactions of 12 proteins, eight of which correspond to genes other than the four mRMR-identified ones and they are all annotated in the Ensemble Biomart database. We list these genes in Table 2 as an extended set of candidate regulatory factors of prostate cancer that are possibly co-acting with the four mRMR-identified surrogate genes.

**Table 2:** **Genes on shortest**-**paths among the four mRMR**-**identified genes**

**Ensembl gene ID**	**Ensembl protein ID**	**Gene symbol**	**Gene function**
ENSG00000091831	ENSP00000206249	ESR1	Hormone receptor; ligand-activated transcription factor
ENSG00000026025	ENSP00000224237	VIM	Cytoskeleton formation and maintenance; organization of cell attachment, migration and signaling
ENSG00000138650	ENSP00000264360	PCDH10	Cadherin-related receptor mediating cell-cell adhesion
ENSG00000152661	ENSP00000282561	GJA1	Gap junction (intercellular communication)
ENSG00000150991	ENSP00000344818	UBC	Ubiquitination
ENSG00000151623	ENSP00000350815	NR3C2	Mineralocorticoid receptor; ligand-dependent transcription factor
ENSG00000163848	ENSP00000353863	ZNF148	DNA-binding transcription factor; regulator in cell growth and apoptosis
ENSG00000198888	ENSP00000354687	MT-ND1	Mitochondrial NADH oxidoreductase; energy metabolism

### Functional annotation of the identified genes

Using the functional annotation tool DAVID, we carried out Gene Ontology (GO) and KEGG Pathway annotation for all the 12 identified genes (including the four mRMR-selected genes and another eight ones traced in the shortest-path PPI network). The results show that many genes are functioning to regulate transcription and/or transcription factor activity (Table 3), echoing the previous finding that genes expressions in (metastatic) prostate cancer are dictated by distinct transcriptional programs [12]. In addition, the genes are also involved in pathways related to steroid hormone receptor activity (Table 3). This is highly consistent with earlier studies that growth of prostate cancer cells is dependent on the male hormone (i.e. androgen) and overly prolonged changes of *in vivo* hormonal level (e.g. androgen deprivation therapy, ADT) causes the emergence of androgen-independent (AI) cancer cells, which result in more malignancy towards advanced or metastatic prostate cancer [24,25]. It is obvious that function of hormone receptor plays a crucial role in prostate cancer progression; and our surrogate gene set captures this reality.

**Table 3:** **GO annotation for genes co**-**identified by mRMR and shortest**-**path analysis**

**Term**	**Genes**	**Count**	%^*^
GO: 0043565 ~ sequence-specific DNA binding	ZNF148, ESR1, NR3C2, MYEF2	4	36. 364
GO: 0030528 ~ transcription regulator activity	ZNF148, UBC, ESR1, NR3C2, MYEF2	5	45. 455
GO: 0010604 ~ positive regulation of macromolecule metabolic process	ZNF148, UBC, ESR1, GJA1	4	36. 364
GO: 0010647 ~ positive regulation of cell communication	UBC, ESR1, GJA1	3	27. 273
GO: 0003700 ~ transcription factor activity	ZNF148, ESR1, NR3C2, MYEF2	4	36. 364
GO: 0003707 ~ steroid hormone receptor activity	ESR1, NR3C2	2	18. 182
GO: 0004879 ~ ligand-dependent nuclear receptor activity	ESR1, NR3C2	2	18. 182
GO: 0005496 ~ steroid binding	ESR1, NR3C2	2	18. 182
GO: 0010628 ~ positive regulation of gene expression	ZNF148, UBC, ESR1	3	27. 273
GO: 0005198 ~ structural molecule activity	VIM, UBC, TUBB6	3	27. 273

^*^“%” refers to the percentage of genes in the total gene set.

Interestingly, the identified predictor set includes genes (e.g. *TUBB6*, *GJA1*) that are annotated by both GO and KEGG pathways as relating to gap junction and regulation of cell communication (Table 4). In fact, it is long recognized that gap junction-mediated intercellular communication is required for cellular normality and breakdown of this communication is a hallmark of cancer [26,27]. Furthermore, earlier studies have shown that intercellular communications and expressions of gap junction-forming proteins are largely reduced or not detected in prostate cancer cells [28,29]. Therefore, our results have faithfully embodied the impact of cell communication dysregulation in prostate cancer.

**Table 4:** **KEGG annotation for genes co**-**identified by mRMR and shortest**-**path analysis**

**Pathway**	**Genes**	**Count**	**%**
hsa04540: Gap junction	TUBB6, GJA1	2	18. 182

## Discussion

In the present study, we applied an informatic approach to identify molecular surrogates underlying the different phases of prostate cancer, which would facilitate deciphering the mechanism(s) of disease progression. The investigation led four genes into our sight — *SLC25A22*, *TUBB6*, *MYEF2* and *PARM1*. Data have shown that these genes ensure the sample classification with the accuracy of more than 70% and the genes are annotated to cancer-relevant functions/pathways. Thus the reasonability of our research is suggested. In the results, two of the four genes (*TUBB6*, *PARM1*) are supported by literature for their roles in prostate cancer; nonetheless, the other two (*SLC25A22*, *MYEF2*) are less known. Therefore, due to the indication of our results, we believe that these two genes may also sustain potentially role(s) during prostate cancer progression and they are worth being focused on for further experimental studies.

*SLC25A22* is named as solute carrier family 25 (mitochondrial carrier: glutamate), member 22. It is involved in the transport of glutamate across the inner mitochondrial membrane (accompanied by H^+^ transportation), which facilitates the malate-aspartate shuttle. The gene has also been validated by another dataset [30]. We hypothesize that the functioning of malate-aspartate shuttle can provide extra energy to cancer cells for gaining the growth advantage against native cells as well as escaping from the original site.

*TUBB6* is a gene encoding a subtype of β-tubulins, the major constituent of microtube, which plays fundamental roles in cell structure maintenance, formation of the mitotic spindle, transportation of chemicals, etc. Furthermore, *TUBB6* is also functionally associated with gap junctional intercellular communication (GJIC). In fact, the respective relationships between both tubulin and GJIC with metastasis of prostate/other cancers have been studied. For instance, the level of tubulin affects the metastasis of colorectal carcinoma cells [31]; and breakdowns of GJIC in a variety of cancer cells correlate their metastatic capacity [32-34]. Moreover, studies have also indicated that *TUBB6* itself is functionally related with the metastasis of various cancers. For instance, in a study using 60 cancer cell lines with different invasion abilities, *TUBB6* is identified as an invasion-associated (IA) gene [35]. Moreover, it is also identified as one of the 38 prognostic gene expression signatures of node-positive breast cancer after systemic adjuvant chemotherapy [36]. In addition, Champine *et al*. have shown that one of the potential mechanisms of BRMS1-mediated metastasis suppression is the suppression of *TUBB6*[37].

*PARM1* is named as prostate androgen-regulated mucin-like protein 1. The gene regulates telomerase protein component 1 (TLP1) expression and telomerase activity, thus enabling certain prostate cells to resist apoptosis. Multiple works have proved that *PARM1* is an important causal gene of prostate cancer [38-40]. It contributes to the immortalization of prostatic cancer cells, which enhances the survival advantage against the neighboring native cells and promotes metastasis. *MYEF2* is myelin expression factor 2, which functions as a transcription repressor of the myelin basic protein (MBP). Our results underline the importance of the gene for prostate cancer, although no direct relationship between *MYEF2* and the cancer had been established yet.

Our findings have provided a concise picture of the metastasis of prostate cancer. According to Valastyan and Weinberg, the metastatic process includes 7 steps [41]: (1) invade locally through surrounding extracellular matrix and stromal cell layers, (2) intravasate through blood vessels, (3) survive during the transportation, (4) arrest at distant organ sites, (5) extravasate into the parenchyma of distant tissues, (6) initially survive in these foreign microenvironments in order to form micrometastases, and (7) re-initiate their proliferative programs at metastatic sites. First, as a cancer cell, energy is its priority (i.e. functional relation with *SLC25A22*). Step (1) and (2) of metastasis need more mobility, for which tubulin will accommodate this task. *PARM1* can enhance the survivability of cancer cells during the transportation and competing with the native cells in the invaded environment.

To investigate the identified surrogate genes further, we have found that they and their co-interacting genes in the PPI network contain existent or potential therapeutic targets for cancers. In fact, Conde-Pueyo *et al*. suggest that *TUBB6* forms a sythetic lethal (SL) association with the cancer-related gene *BUB1*, speculating that treatments targeting the tubulin gene should be more efficient in cancers where *BUB1* is mutated [42]. Moreover, tubulins are existent targets of anti-cancer drugs, e.g. Paclitaxel and vinca alkaloids (e.g. Vincristine and Vinblastine) in various cancers (including prostatic). The drugs disrupt the formations of microtubules/mitotic spindles and hence inhibit the proliferations and metastases of cancer cells [43,44]. *PARM1* is part of the Golgi apparatus that is androgen-responsive, and researches demonstrate that the Golgi apparatus embody new mechanisms of the androgen receptor (AR)-mediated signaling and they are useful biomarkers for prostate cancer diagnosis/prognosis [45]. Moreover, Golgi-targeting drugs have been shown to be effective in both androgen-dependent/-independent prostate cancers [45]. Given this foreground, *PARM1* may have the potential of a therapeutic target for prostate cancer. It is also noteworthy that although the other two genes we identified (*SLC25A22* and *MYEF2*) do not possess direct therapeutic utilities at present, they may somehow implicate theoretical clues for cancer therapies. In fact, members of the SLC25A family (e.g. *SLC25A4*/*5*/*6*) are existent drug targets for the treatments of bone metastases in breast cancer and metastatic bone disease [46]; since prostate cancer also exhibit bone metastasis [3], *SLC25A22* may be worth being examined for potential relations to the metastatic properties of prostate cancer. Meanwhile, *MYEF2* has been characterized as a downstream target modulated by the Wnt/β-catenin pathway; since inhibition of Wnt/β-catenin signaling suppresses a number of cancers (e.g. multiple myeloma, colorectal cancer, etc.) [47], the genes regulated by Wnt/β-catenin may provide insights into the mechanisms of cancer developments and therapies.

Furthermore, in the PPI partners co-identified with the surrogate genes, *ESR1* (estrogen receptor 1) is a widely known therapeutic target (for selective modulators, e.g. Raloxifene, Tamoxifen, etc.) in breast cancer in female [48]; however, its roles in the prostate cancer in male have not been revealed. In addition, *NR3C2* (also known as the mineralocorticoid receptor, MR) belongs to the same family with the androgen receptor (AR, also known as *NR3C4*). Since they are co-interacting with *PARM1* and *MYEF2* (Figure 2), *ESR1* and *NR3C2* may also participate in prostate cancer along with the existent/potential targets. Moreover, other co-identified genes via PPI are also informative for cancer researches. *PCDH10* (protocadherin 10) is a potential target for demethylation drugs to achieve its reactivation, which may facilitate the therapies of a wide variety of cancers (e.g. cervical, gastric, colorectal, breast cancers and leukemias) [49,50]. *ZNF148* (also known as *ZBP*-*89*) regulates cell grwoth and apoptosis, having crucial roles in the developments of many cancers (e.g. gastric, colorectal, breast cancers). It is a potential target in cancer therapy as experiments show that *ZNF148* is a tumor suppressor capable of enhancing the killing effects of several anti-cancer drugs [51]. Therefore, we hypothesize that these therapeutic targets may also have biological roles in prostate cancer, or prostate cancer may have regulators that are in common with other cancers. In addition, *VIM* (vimentin) is characterized as an invasion/metastasis factor in tumor cells, which is transcriptionally regulated by *HIF*-*1*[52]. *GJA1* (gap junction protein, alpha 1) involves in gap junction (GJIC), which plays important roles in cancer progression/metastasis. *MT*-*ND1* encodes a mitochondrial oxidoreductase (NADH dehydrogenase 1) involving in energy metabolism, which is intuitively crucial to cancer development. In fact, both gap junctions and mitochondria are emerging as therapeutic targets in cancers nowadays [53,54].

The previous work of Chandran *et al*. has discovered hundreds of genes with differential expression profiles [11]. In order to decipher the disease more concisely, we adopt the mRMR algorithm, which can provide results with the largest parsimony. Our results have showed that prostate cancer samples can be classified with only four genes, indicating that although the cancer is a complex disease with hundreds of differentially-expressed genes, these four genes may be the primary surrogates for the mechanism (s) underlying the different cancer phases. Moreover, these four genes (along with their PPI partners) turn out having mechanistic/therapeutic implications in the prostatic or other cancers. Hence, in order to characterize the development of prostate cancer (especially metastasis) and investigate the molecular mechanism (s), these genes could firstly be focused on in further functional experiments.

## Conclusion

In all, we have characterized a small-sized predictor gene set for classification of prostate cancer phases. Our results support the roles for specific genes involved in cell communication, hormone-receptor mediated pathways, and transcription regulation in (metastatic) prostate cancer. To our knowledge, the gene set we computed is of the minimal size that can rationally characterize prostate cancer phases; thus we hypothesize that these genes potentially play important roles in the molecular mechanisms of prostate cancer development. Furthermore, due to the small size, our predictor gene set can be a suitable candidate list for forthcoming functional experiments; meanwhile, it might possess potential value for other relevant studies (e.g. drug target selection).

## Abbreviations

mRMR: Minimum redundancy - maximum relevance; IFS: Incremental feature selection; PPI: Protein-protein interaction; NT: Normal tissue; PT: (tissue-confined) Primary tumor; MT: Metastatic tumor; SLC25A22: Solute carrier family 25 member 22; TUBB6: Tubulin, beta 6 class V; PARM1: Prostate androgen-regulated mucin-like protein 1; MYEF2: Myelin expression factor 2; PPARγ: Peroxisome proliferator-activated receptor gamma; BRMS1: Breast cancer metastasis suppressor 1.

## Competing interests

The authors declare that they have no competing interests.

## Authors’ contributions

Conceiving the research: RL, XD, CM and LL. Data analysis: RL, XD and CM. Manuscript writing: RL, XD, CM and LL. All authors read and approved the final manuscript.

## Supplementary Material

Additional file 1: Table S1 Prediction accuracies for each individuals in IFS. The file includes detailed prediction data for the normal condition (denoted by“A”), primary tumor (“B”) and metastatic tumor (“C”). “Prediction 1 - 3” have the same meaning as in Figure 1, and the prediction statuses are shown. The file is in the format of electronic data sheet (Microsoft Excel *.xlsx). Click here for file

Additional file 2: Table S2 Expression profiles of the four mRMR-selected genes. The file shows expression levels of the four mRMR-selected genes in the three class of samples. The file is in the format of Microsoft Excel *.xlsx. Click here for file
